# Preparation and Characterization of Controlled-Release Floating Bilayer Tablets of Esomeprazole and Clarithromycin

**DOI:** 10.3390/molecules27103242

**Published:** 2022-05-18

**Authors:** Muhammad Israr, Nicola Pugliese, Arshad Farid, Shakira Ghazanfar, Alessandro Di Cerbo, Muhammad Muzammal, Abdulhakeem S. Alamri, Syed Mohammed Basheeruddin Asdaq, Ashfaq Ahmad, Kamran Ahmad Khan

**Affiliations:** 1 Gomal Centre of Pharmaceutical Sciences, Faculty of Pharmacy, Gomal University, D. I. Khan 29050, Pakistan; khanmuhammadisrar011@gmail.com; 2 Department of Veterinary Medicine, University of Bari, 70010 Valenzano, Italy; nicola.pugliese@uniba.it; 3 Gomal Centre of Biochemistry and Biotechnology, Gomal University, D. I. Khan 29050, Pakistan; mustafamuzammal1@yahoo.com; 4 National Institute for Genomics Advanced Biotechnology, National Agricultural Research Centre, Park Road, Islamabad 45500, Pakistan; shakira_akmal@yahoo.com; 5 School of Biosciences and Veterinary Medicine, University of Camerino, 62024 Matelica, Italy; 6 Department of Clinical Laboratory Sciences, Faculty of Applied Medical Sciences, Taif University, Taif 26432, Saudi Arabia; a.alamri@tu.edu.sa; 7 Centre of Biomedical Sciences Research (CBSR), Deanship of Scientific Research, Taif University, Taif 26432, Saudi Arabia; 8 Department of Pharmacy Practice, College of Pharmacy, AlMaarefa University, Dariyah 13713, Saudi Arabia; sasdag@mcst.edu.sa; 9 Department of Pharmacy, University of Swabi, Swabi 23430, Pakistan; ashfaqahmad@uoswabi.edu.pk

**Keywords:** clarithromycin, esomeprazole, floating bilayer tablets

## Abstract

Controlled-release effervescent floating bilayer tablets reduce dosage frequency and improve patient compliance with enhanced therapeutic outcomes. Generally, two different tablets of clarithromycin and esomeprazole, respectively, are given for the treatment of *Helicobacter pylori* infection and it might be worth incorporating both in a single tablet. In the current study, controlled-release floating bilayer tablets of clarithromycin and esomeprazole (F1–F4) were developed with different rates of polymeric materials by a direct compression method. During the formulation, Fourier-transform infrared spectroscopy (FTIR) analysis was performed for possible interactions between drugs and excipients. No interactions between drugs and excipients were noted. Moreover, the bilayer tablets’ thickness, diameter, friability, hardness, weight variation, dissolution, and percent purity were found within the acceptable limits. The floating lag time and total floating time of all formulations were found to be < 25 s and 24 h, respectively. The release of both the clarithromycin and esomeprazole started at the same time from the controlled-release floating bilayer tablets by anomalous non-Fickian diffusion, and the polymeric materials extended the drug release rate up to 24 h. In the case of F1, the results approached ideal zero-order kinetics. The dissolution profiles of the tested and reference tablet formulations were compared, but no significant differences were observed. It can be concluded that such controlled-release effervescent floating bilayer tablets can be efficiently used in clinical practice to reduce dosage frequency and increase patient compliance with continuous drug release for 24 h, which ultimately might enhance therapeutic efficacy.

## 1. Introduction

Floating drug delivery systems are designed to deliver drugs with low intestinal solubility or poor stability into the stomach by floating over the gastric fluid due to their low-density [1,2]. These dosage forms are embedded within tablets made of hydroxypropyl cellulose and sodium and citric bicarbonate, two gas-generating agents that, when coming into contact with gastric fluid, generate carbon dioxide, which promotes the floating of the tablets [3].

The reported optimum ratio of citric and sodium bicarbonate for carbon dioxide generation is 0.76:1. The aforementioned tablets may be single-layered or bi-layered; in this latter case, the gas-generating agents are in one layer and the drug is added in the sustained-release portion layer [1]. Moreover, they are made of either natural polymers (Xanthan gum, Gellan gum chitosan, Guar gum, sodium alginate) or synthetic polymers (Eudragit, Ethylcellulose, hydroxypropyl cellulose) [4].

Drugs with a shorter half-life (2–6 h), better bioavailability, dissociation constant >2.5, partition constant >1, and stability in an acidic environment are suitable for the inclusion in floating drug delivery systems [5]. These systems allow a controlled drug release for an extended period, keeping the dose at the absorption site and therefore improving the bioavailability [6].

*Helicobacter pylori* management with proton pump inhibitors, e.g., esomeprazole, clarithromycin 500 mg, amoxicillin 1 g, or metronidazole 400 or 500 mg, is known to lower the risk of gastroduodenal ulcers and avoid its relapse. Despite that, a 14-day regimen may be more beneficial than a 7-day one, the optimum effective period of therapy is still a matter of debate [7,8].

Esomeprazole is a proton pump inhibitor for the management of gastroesophageal reflux disease (GERD) and the recovery and maintenance treatment of erosive esophagitis, but it is also a component of the triple-drug regimen for the *Helicobacter pylori* infection [9].

On the other hand, clarithromycin is a broad-spectrum antibiotic belonging to the macrolides class with a molecular weight of 747.95 Dalton, a pKa value of 8.99, a half-life of ~4 h, soluble acetonitrile, ethanol, and acetone, slightly soluble in methanol, and poorly soluble in water (0.33 mg/L) [10]. Clarithromycin-based floating matrix tablets have been developed using hydroxypropyl cellulose K15M, hydroxypropyl cellulose K4M, hydroxypropyl cellulose, and hydroxyethylcellulose, obtaining an in vitro controlled release of the antibiotic [11]. Moreover, floating matrix tablets have also been developed using a natural polymer, e.g., pomegranate peels powder, achieving a floating time of 5 h and a 98.67% of drug release [12].

In the current research, new controlled-release floating bilayer tablets of esomeprazole and clarithromycin were prepared and assessed for various physicochemical characteristics, such as a dimensional test, friability, hardness, and weight variation, and in vitro evaluation.

## 2. Materials and Methods

### 2.1. Materials

Clarithromycin (Wilson Pharmaceutical, Islamabad, Pakistan), Esomeprazole (Wilson Pharmaceutical, Islamabad, Pakistan), Magnesium stearate (BDH Chemical limited, Poole, UK), Carbopol^®^ 934 P (Lubrizol, Wickliffe, OH, USA), Sodium bicarbonate (BDH Chemical limited, Poole, UK), HCl 35% (Merck, Darmstadt, Germany), Avecil 102 and Talc (Wilson’s Pharmaceutical Islamabad), Eudragit^®^ RS 100 (Rohm GMBH, Darmstadt, Germany), single-punch tablet machine (Erweka-AR-400 made in West Germany), Digital electronic balance (AX120, Shimadzu, Japan), UV–visible spectrophotometer (UV-1601, Shimadzu, Japan), USP apparatus type-I (Hamburg, Germany), Friabilator (Erweka TA3R Apparatus, Langen, Germany), Fourier-transform infrared spectrophotometer (LI600300 spectrum Two Lita, Llantrisant, UK), Hardness tester (Erweka TB24 Apparatus, Germany).

### 2.2. Tablets Formulation

A total of 144 clarithromycin and esomeprazole controlled-release floating bilayer tablets were formulated to allow a constant amount of drug release, while polymeric materials differed depending on the formulation, as shown in Table 1. In each formulation, a gas-generating agent was also added to bring the tablet to the surface in a simulated gastric medium (0.1 N HCl).

### 2.3. Flow Properties

Flow parameters such as Hausner ratio, compressibility index, and angle of repose were determined according to standard procedures for powder mixtures formulations [13].

### 2.4. Angle of Repose

The angle of repose is the angle between the pile of powder and the horizontal plan and measures frictions between powder or granules particles [13]. The angle of repose was determined for pure drugs and all the formulations were prepared manually using the funnel and cone method. Initially, pure drugs were passed separately through a funnel fixed above a petri dish, and the height (h) of the powder heap and petri dish diameter were calculated according to the formula: θ = tan^−1^ h/r.

### 2.5. Compressibility Index and Hausner’s Ratio

Bulk and tapped densities were used for the determination of compressibility index and Hausner ratio. The powder was added manually in a graduated cylinder and the surface was leveled to note bulk volume and then tapped 100 times to attain the tapped volume. Bulk density and tapped density were determined from volume values, while compressibility index and Hausner ratio were calculated from bulk density and tapped density [13].

### 2.6. FTIR Analysis

Once all formulations of drugs were formulated, an FTIR analysis was performed according to the standard procedure for possible interaction between drugs and excipients. Pure drug and formulation mixtures were examined by measuring wavenumbers of 4000–400 cm^−1^ at a room temperature [14] to obtain FTIR spectra of drugs and drugs along with excipients and were checked for possible interactions.

### 2.7. Tablets Preparation

Tablets were prepared by the direct compression method [15]. Initially, all ingredients were weighed, then geometrically mixed with polythene bags and lubricants, and finally passed twice through 60-mesh sieves for thorough mixing. Manually, the mixture of the clarithromycin layer was put into the die cavity (13.15 mm) of a single-punch tableting machine and compressed. Then, the esomeprazole layer was added and compressed into bilayer tablets individually. Pilot batches of 140 tablets were prepared for each type of formulation for further characterization. The hardness of tablets was kept constant at 5–10 kg/cm^2^.

### 2.8. Physical Quality Control Tests

Various physical quality control tests were performed according to standard procedures [15]. General appearance, shape, thickness, diameters, hardness, friability, and weight variation test.

### 2.9. General Appearance and Shape

The general appearance of tablets was observed with naked eyes and was found elegant. The shape was checked with a magnifying lens.

### 2.10. Diameter and Thickness

The diameter and thickness of 10 tablets randomly selected from each batch were determined using a Vernier caliper. Each tablet’s diameter was recorded as mean ± SD [16].

### 2.11. Hardness

Hardness was evaluated with a hardness tester for 10 tablets selected for each batch randomly and average hardness was calculated as mean ± SD in units of kg/cm^2^ [17]. In the hardness tester, a tablet was placed and breaking force was noted from the recorder, and then the average hardness of 10 tablets was calculated.

### 2.12. Friability Test

Friability was determined on 10 tablets with a friabilator that rotated at 25 rpm for a rate of 4 min and then, after 100 revolutions, tablets gained weight. Initial weight was measured as W1 and final as W2. Then, friability was calculated as percent and mean ± SD [11].

% Friability = (W1 − W2/W1) × 100(1)

### 2.13. Weight Variation Test

Ten tablets of each batch were weighed, and the average weight was noted as mean ± SD [18].

### 2.14. Floating Behavior

#### Swelling Behavior

When tablets came in contact with water, polymeric materials started swelling in the tablets; then, all tablets from each batch were taken and placed in 0.1 N HCl 25 mL at 37 ± 0.5 °C and stirred at 25 rpm. Weighed tablets (W_o_) were wiped gently with filter paper to remove water on the surface and reweighed (W_t_) [19]. Then, water uptake or swelling index was calculated as follows:

Water uptake = (W_t_ − W_o_/W_o_) × 100 (2)

### 2.15. Tablet Density

The floating density of tablets is a very important parameter to be considered for the floating behavior of tablets, as a tablet can float when its density is less than 1.004 g/cm^3^. Tablets’ volume was calculated according to the formula (v = πr^2^h), and tablets’ mass was determined accordingly. The density of formulations was determined for each batch according to the formula (ρ = m/v) [11].

### 2.16. Buoyancy

These tablets had gas-generating material and when came in contact with water, CO_2_ was produced inside the tablets that floated on the surface. USP Method-II (paddle method) was used to determine tablet buoyancy/floating. A total of 0.1 N HCl (900 mL) was added to the flask of the apparatus with a rotation speed of 50 rpm and the temperature was maintained at 37 ± 0.5 °C for 24 h. Tablets’ total floating time and floating lag time were calculated [20].

### 2.17. Chemical Assay

A chemical assay for all formulations was performed according to a validated method [21]. Randomly, ten tablets were taken from each batch and crushed to powder, and then powder equivalent to 250 mg of Clarithromycin was taken into a 100 mL flask containing 0.1 N HCl. A total of 5 mL of this solution was taken in a 100 mL volumetric flask and diluted with 95 mL of 0.1 N HCl and filtered. Samples were analyzed spectrophotometrically at 276 nm. Powder equivalent to 20 mg of Esomeprazole was taken and added to 100 mL 0.1 N HCl solution and dissolved. A total of 5 mL of this solution was taken in a 100 mL volumetric flask, diluted with 95 mL of 0.1 N HCl, and filtered. Samples were analyzed spectrophotometrically at 284 nm [14,22].

### 2.18. Drug Release Study

USP Method-II was used for the dissolution test, which was carried out initially in the 0.1 N HCl solutions for 6 h and then using the phosphate buffer (pH 7.4) for the next 18 h. The experiments were performed in a pharma test dissolution apparatus (Hamburg, Germany), with a rotation speed of 75 rpm and a dissolution medium temperature of 37 ± 0.5 °C. At specified time intervals, 5 mL samples were taken and a replacement volume of dissolution medium was added. After filtration, samples were separately spectrophotometrically analyzed at a fixed wavelength for clarithromycin (276 nm) and esomeprazole (284 nm). The drug release was calculated from the respective standard curve of each drug [23].

### 2.19. Drug Release Kinetic

First-order (ln (100 − W) = ln100 − K_2_t), zero-order (W = K_1_t), and power-law kinetic model (M_t_/M_∞_ = Kt^n^) [24] were used to obtain the drug release mechanism from cumulative drug release data of all the formulations.

### 2.20. Dissolution Profile Comparison

Differences and similarities between reference and tested formulations were determined by applying the difference and similarity factor [25].

### 2.21. Statistical Analysis

Data were analyzed using GraphPad Prism 7 software (GraphPad Software, Inc., La Jolla, CA, USA). All data are presented as the means ± standard deviation (SD) and were first checked for normality using the D’Agostino-Pearson normality test.

The comparison between dissolution profiles of tested formulations and reference formulation comparison was performed using a Paired *t*-test. A * *p* < 0.05 was considered significant.

## 3. Results and Discussion

### 3.1. Flow Characteristics

The flow properties to obtain an easy-to-produce product were determined according to the standard procedure. All tablet formulations were found to have good to excellent flow characteristics as the values of the angle of repose ranged from 26.4 ± 0.18 to 30.0 ± 0.06°, the compressibility index from 11.28 ± 0.02 to 13.16 ± 0.01%, and the Hausner ratio from 1.12 ± 0.02 to 1.14 ± 0.18 [22,26,27,28,29].

The results of the flow characteristics are summarized in Table 2.

### 3.2. FTIR Study

**Figure 1:**
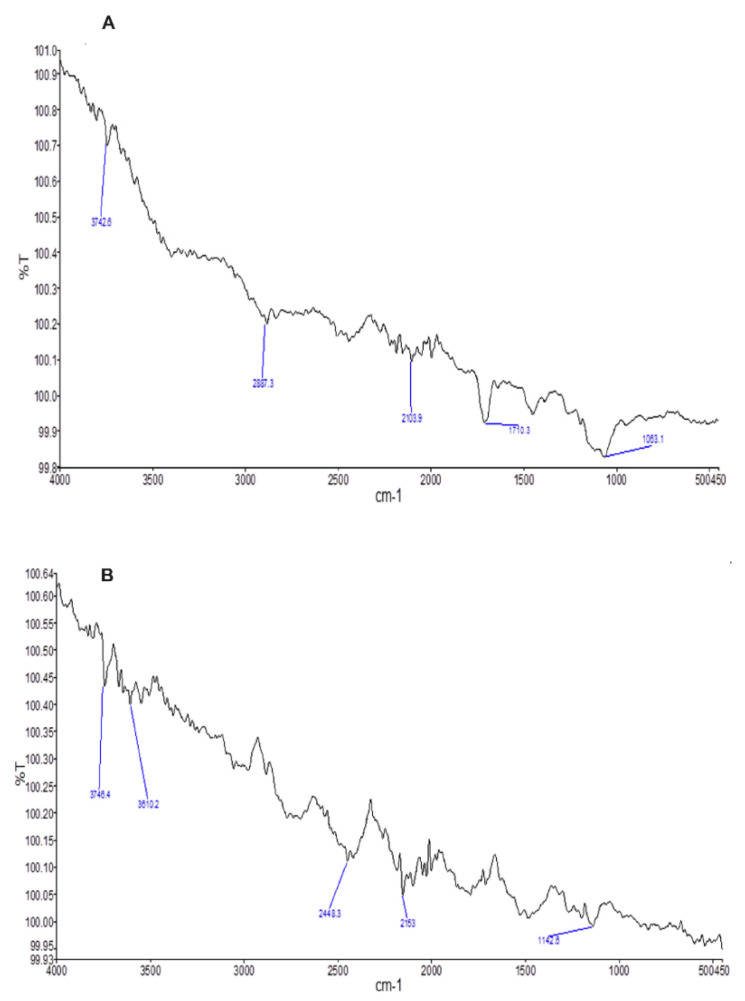
Graphical representation of (**A**) clarithromycin and (**B**) formulation F1 formulation FTIR spectra.

To check the computability of the drug with the excipients, an FTIR analysis was performed according to the specified protocol. Clarithromycin (Active Pharmaceutical Ingredient) (Figure 1A) showed a specified spectrum similar to that of its formulation (F1) (Figure 1B), thus indicating the lack of any interaction.

**Figure 2:**
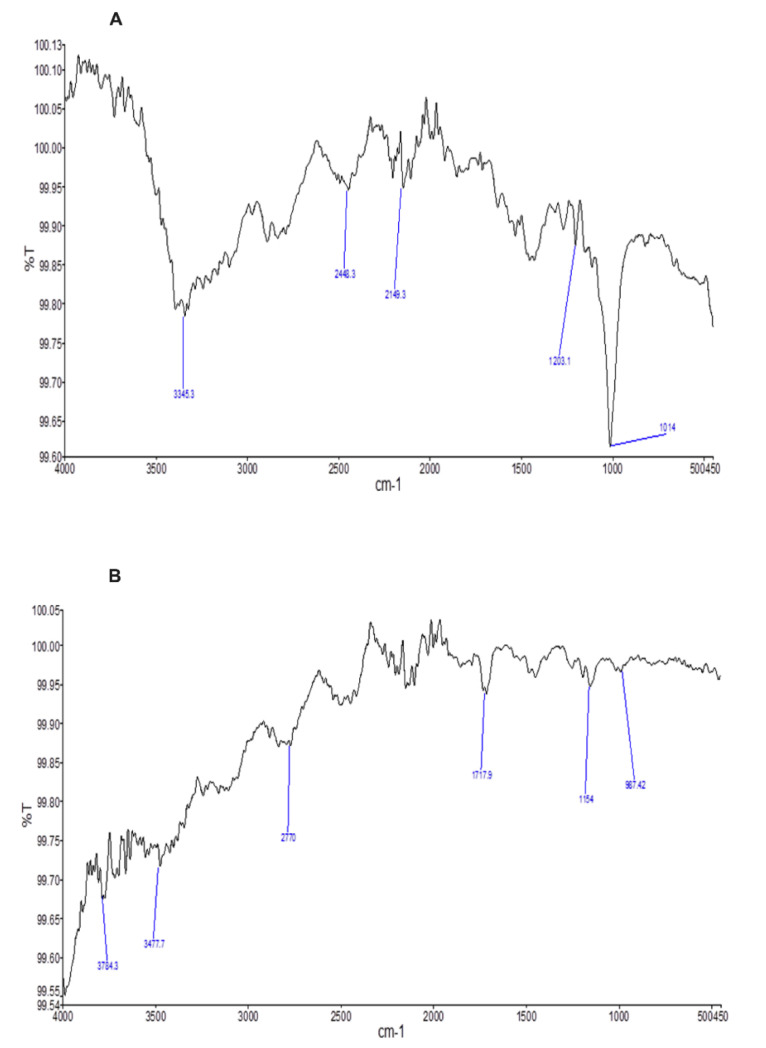
Graphical representation of (**A**) esomeprazole and (**B**) formulation F1 formulation FTIR spectra.

Similarly, esomeprazole (API) (Figure 2A) and its formulation (F1) spectra (Figure 2B) shared similarity and the functional groups’ positions were intact in both spectra.

The main peaks in the clarithromycin spectrum were OH/NH (3742.6 cm^−1^), CH2 asymmetric (2881.3 cm^−1^), C≡C stretching (2103.8 cm^−1^), C=C stretch (1710.3 cm^−1^), and C–C stretch (1063 cm^−1^), while the clarithromycin formulation peaks were OH/NH (3746.4 cm^−1^ and 3610.2 cm^−1^), C–H (2448.3 cm^−1^), C≡C stretching (2153 cm^−1^), and C–C (1142 cm^−1^).

The main peaks in the esomeprazole spectrum were NH-stretch (3345 cm^−1^), CH-stretch (2448.3 cm^−1^), CH (2149 cm^−1^), C–O stretch (1203 cm^−1^), and C–O stretch (1014 cm^−1^), while the esomeprazole formulation peaks were OH/NH (3784.3–3477.3 cm^−1^), OH-stretch (2770–1717.9 cm^−1^), and C–O stretch (1154 cm^−1^).

Our results were in agreement with previous studies conducted using an FTIR analysis [30].

### 3.3. Physical Quality Control Tests

Physical quality-control tests were applied according to the specified protocols. All the tables appeared flat and smooth. The controlled-release floating bilayer tablets’ thickness ranged from 3.530 ± 0.11 to 3.560 ± 0.139 mm, the diameter from 13.151 ± 0.0748 to 13.155 ± 0.0057 mm, the hardness from 6.56 ± 0.268 to 6.80 ± 0.189 kg/cm^2^, the friability from 0.02 ± 0.081 to 0.06 ± 0.262, and the weight from 671.6 ± 0.98 to 674.0 ± 1.45 mg.

These results were in agreement with other authors [31] and have been reported in Table 3.

### 3.4. Swelling Behavior

**Figure 3:**
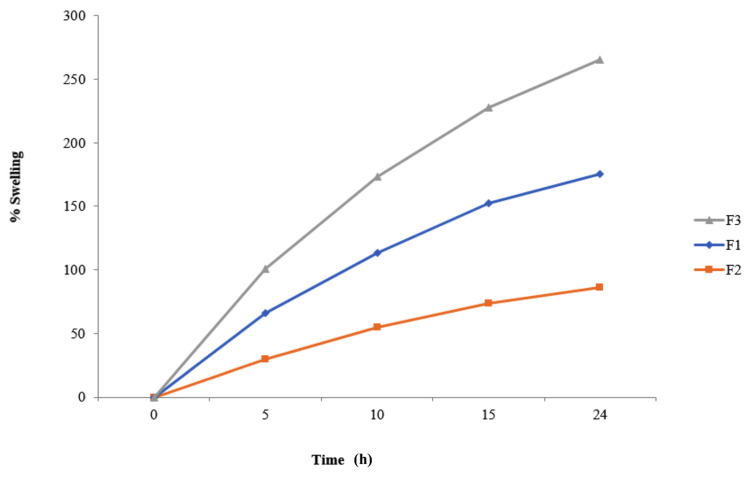
Graphical representation of the swelling behavior of F1, F2, and F3 formulations.

According to the literature, all tablets must have a density lower than that of the gastric fluid (1.004 g/cm^3^) to float over the gastric content [32]. In this sense, the density of all controlled-release floating tablets was less than (1.004 g/cm^3^). The water uptake ratio to the swelling was shown by the polymeric material that retained some water. Our current formulations swelled up to 89.90% of the original size due to the hydrophilicity of the polymeric materials, particularly Carbopol 934 (Figure 3) [33].

### 3.5. Floating Behavior and Density

All the controlled-release bilayers showed good floating behavior due to the presence of a floating agent, e.g., sodium bicarbonate, which started floating once it came into contact with the simulated gastric fluid (0.1 N HCl pH 1.2 at 37 ± 0.5 °C). The floating lag time ranged from 21 to 25 s. The total floating time was noted for 24 h and all formulations showed a total floating time >24 h. The density was found to be less than 1.004 g/cm^3^.

The results are shown in Table 4 and are in perfect agreement with other authors [11].

### 3.6. Drug Release

**Figure 4:**
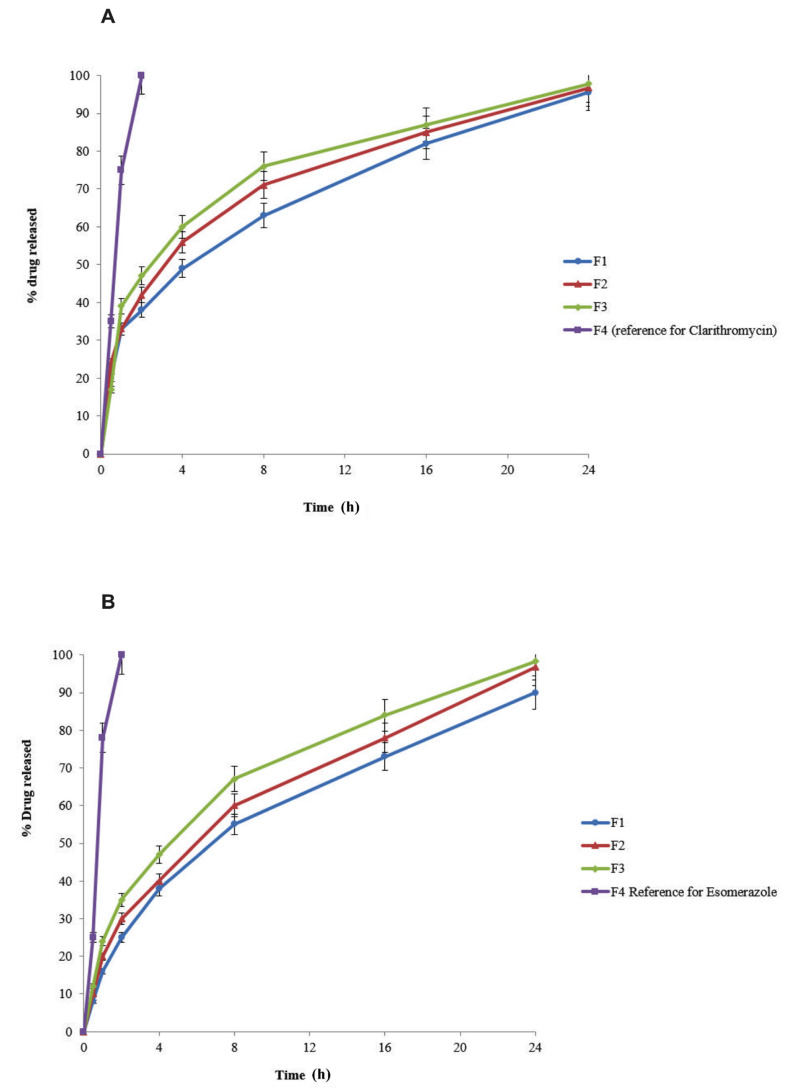
Graphical representation of (**A**) clarithromycin and (**B**) esomeprazole release profiles from controlled-release floating bilayer tablets and reference formulations.

Both clarithromycin and esomeprazole were released for 24 h from their respective polymeric controlled-release floating bilayer tablets (F1–F3), thus extending the drug release rate (Figure 4). In particular, F1 released 95.55% of the clarithromycin and 90% of the esomeprazole, F2 released 96.76% and 96.78%, and F3 97.86% and 98.34%.

The drug release rate was extended by polymers, but because Eudragit RS 100 is a pH-dependent polymer, it affected the release by changing the pH from acidic to alkaline. F1 (Carbopol^®^ 934P and Eudragit RS100) reduced the drug release with respect to F2 (only Eudragit^®^ RS 100), while F3 (Carbopol^®^ 934P) delayed the release.

We hypothesized that the extended drug release rate could be ascribed to the polymer Carbopol^®^ 934P, as it was also reported in the previous work that Carbopol^®^ 934P extended the Tramadol release rate when used as a rate-controlling agent [33].

### 3.7. Content Uniformity

The content uniformity for each batch was determined, and it was noted that the clarithromycin percent purity ranged from 98.89 ± 0.087 to 101.23 ± 0.026% and that of the esomeprazole ranged from 99.54 ± 0.049 to 101.99 ± 0.023% (Table 5). These results were found within acceptable limits according to the United States and European Pharmacopeia [34].

### 3.8. Drug Release Mechanisms

The drug release mechanisms were determined by applying first-order, zero-order, and power-law kinetic models (Table 6). The results of the first-order kinetic model showed that the model was best fitted in F4 as it was an immediate release formulation, while the zero-order kinetic model was followed by the F1 to F3 formulations and F4 did not follow the zero-order kinetic model for both the clarithromycin and esomeprazole, and these results are in similarity with other authors [24]. The clarithromycin release data were fitted into the power-law kinetic model and the R^2^ (regression equation) values ranging from 0.9189 to 0.9896 showed linearity in kinetics. Meanwhile, the *n* (drug release exponent) values ranged from 0.7863 to 0.9356, demonstrating that the drug was released by anomalous non-Fickian diffusion. The clarithromycin released from F1 with an *n* value of 0.9356 was closer to 1, which indicated that this formulation was approaching ideal zero-order kinetics. The reference standard *n* value was 0.3987, indicating that it did not follow a power-law kinetic. In the case of the esomeprazole, the R^2^ value ranged from 0.9645 to 0.9854, showing linearity, while the *n* value ranged from 0.7234 to 0.9653, showing that the drug was released by anomalous non-Fickian diffusion. Formulation F1 released the esomeprazole with an *n* value near 1 showing an ideal kinetic, i.e., zero-order. Its reference formulation *n*-value was 0.412, indicating that it did not follow a power-law kinetic. These results fell within the acceptable range (*n*-value 0.5 to 1) [24], as observed for losartan potassium [24].

### 3.9. Dissolution Profile Comparison

The dissolution profiles of the tested formulations (F1, F2, and F3) and reference formulation (F4) were compared separately by applying the difference factor f1 and similarity factor f2 (Table 7). The difference factor value ranged from 24.453 to 38.431, while the f2 values ranged from 18.39 to 43.532. These results were not within the acceptable limit of f1 and f2, 1–15 and 50 to 100, respectively [25].

### 3.10. Test Statistics

The results showed that there was no similarity between the tested formulations profiles and the reference profile (Table 8).

## 4. Conclusions

In our research, the effervescent controlled-release bilayer tablets of clarithromycin and esomeprazole were successfully developed and characterized for various parameters. In a buoyancy study, it was found that tablets started floating in a very short time, about 20–24 s, and floated for 24 h. An in vitro dissolution was performed to know about drug release patterns. The polymeric material extended the drug release rates up to 24 h and might be used as a once-a-day (OD) tablet. The drug was released by anomalous non-Fickian diffusion or case-II zero-order kinetics almost near to ideal zero-order kinetics. The percent purity of both drugs was within the USP acceptable limits. The dissolution profile of the tested and reference tablets and the f1 and f2 values were not in the acceptable range. The statistic was applied to the dissolution profiles of the tested and reference formulations, and no similarity was noted between the dissolution profiles.

These polymeric bilayer floating tablets might lead to patient compliance, reduce side effects, and minimize dosage frequency with zero-order drug release kinetics.

## Figures and Tables

**Table 1:** Composition of tablets.

Drugs and Excipients		CR Bilayer Floating Tablets			IR Bilayer Tablet
		F1	F2	F3	F4
Clarithromycin layer	Clarithromycin	250 mg	250 mg	250 mg	250 mg
	Eudragit^®^ RS 100	76 mg	152 mg	---------	---------
	Carbopol 934 P	76 mg	---------	152 mg	--------
	Talc	11.8 mg	11.8 mg	11.8 mg	11.8 mg
	Mg Stearate	5.4 mg	5.4 mg	5.4 mg	5.4 mg
	Avecil 102	40.8 mg	40.8 mg	40.8 mg	192.8 mg
	Sodium bicarbonate	80 mg	80 mg	80 mg	80 mg
Esomeprazole layer	Esomeprazole	20 mg	20 mg	20 mg	20 mg
	Eudragit^®^ RS 100	30 mg	60 mg	---------	-------
	Carbopol 934 P	30 mg	---------	60 mg	-------
	Talc	2.6 mg	2.6 mg	2.6 mg	2.6 mg
	Mg Stearate	1.3 mg	1.3 mg	1.3 mg	1.3 mg
	Avecil 102	14.1 mg	14.1 mg	14.1 mg	74.1 mg
	Sodium bicarbonate	32 mg	32 mg	32 mg	32 mg

**Table 2:** Results of flow characteristics.

Formulation	Angle of Repose	Hausner Ratio	Compressibility Index (%)
F1	28.3 ± 0.16	1.124 ± 0.02	11.64 ± 0.03
F2	26.4 ± 0.18	1.132 ± 0.09	13.16 ± 0.01
F3	27.9 ± 0.15	1.143 ± 0.18	11.28 ± 0.02
F4	30.0 ± 0.06	1.138 ± 0.03	12.27 ± 0.04

**Table 3:** Results of physical quality control tests.

Code	Thickness (mm)	Diameter (mm)	Hardness (kg/cm^2^)	Friability (%)	Weight Variation (mg)
F1	3.56 ± 0.03	13.15 ± 0.07	6.56 ± 0.26	0.04 ± 0.12	673.9 ± 0.38
F2	3.53 ± 0.10	13.15 ± 0.07	6.80 ± 0.18	0.02 ± 0.08	671.6 ± 0.98
F3	3.53 ± 0.11	13.15 ± 0.01	6.57 ± 0.54	0.05 ± 0.32	674.0 ± 1.45
F4	3.56 ± 0.13	13.15 ± 0.01	6.75 ± 0.21	0.06 ± 0.26	671.9 ± 0.87

**Table 4:** Floating behavior of CR bilayer floating tablets.

Formulation	Tablet Density (g/cm^3^)	Floating Lag Time (Seconds)	Total Floating Time (Hours)
F1	0.93	20	>24.0
F2	0.91	24	>24.0
F3	0.95	20	>24.0
F4	Immediate release formulation taken as reference		

**Table 5:** Content uniformity of Clarithromycin and Esomeprazole.

Formulation	Content Uniformity (Clarithromycin)	Content Uniformity (Esomeprazole)
F1	98.89 ± 0.087	100.83 ± 0.018
F2	99.96 ± 0.028	99.54 ± 0.049
F3	100.73 ± 0.031	101.99 ± 0.023
F4	101.23 ± 0.026	100.77 ± 0.044

**Table 6:** The release mechanism of Clarithromycin and Esomeprazole.

Formulations	First-Order Kinetic Model	Zero-Order Kinetic Model	Power Law			
	R^2^	R^2^	K ± SD	R^2^	N	Release Mechanism of Clarithromycin
F1	0.568	0.991	0.0012 ± 0.005	0.9189	0.9356	AND
F2	0.619	0.988	0.0036 ± 0.08	0.9896	0.8654	AND
F3	0.731	0.980	0.0056 ± 0.03	0.9869	0.7863	AND
F4	0.993	0.457	0.0001 ± 0.02	0.6776	0.3987	Does not follow a power law
**Formulations**	**First-Order Kinetic Model**	**Zero-Order Kinetic Model**	**Power Law**			
	**R^2^**	**R^2^**	**K ± SD**	**R^2^**	**N**	**Release Mechanism of Esomeprazole**
F1	0.573	0.994	0.0145 ± 0.02	0.9645	0.9653	AND
F2	0.645	0.991	0.0238 ± 0.03	0.9854	0.8654	AND
F3	0.765	0.983	0.1893 ± 0.16	0.9812	0.7234	AND
F4	0.992	0.463	0.0002 ± 0.12	0.7451	0.412	Does not follow power law
AND = Anamolous non-Fickian diffusion						

**Table 7:** Dissolution patterns comparisons.

Test vs. Reference	f1	f2
Clarithromycin profile comparisons		
F1 vs. F4 (reference)	32.76	38.56
F2 vs. F4 (reference)	36.45	39.09
F3 vs. F4 (reference)	34.32	43.53
Esomeprazole profile comparisons		
F1 vs. F4 (reference)	38.43	18.39
F2 vs. F4 (reference)	33.65	23.34
F3 vs. F4 (reference)	24.45	22.98

**Table 8:** Paired *t*-test results.

Test vs. Reference	*p*-Value for Clarithromycin	*p*-Value for Esomeprazole
F1 vs. F4 (reference)	7.2134	5.1823
F2 vs. F4 (reference)	6.4453	6.2434
F3 vs. F4 (reference)	4.5446	4.3465

## Data Availability

Requests to access the datasets should be directed to the corresponding authors.

## Sample Availability

Samples of the compounds are not available from the authors.
